# Investigation of Structures, Stabilities, and Electronic and Magnetic Properties of Niobium Carbon Clusters Nb_7_C_n_ (n = 1–7)

**DOI:** 10.3390/molecules29081692

**Published:** 2024-04-09

**Authors:** Hui-Fang Li, Huai-Qian Wang, Jia-Ming Zhang, Lan-Xin Qin, Hao Zheng, Yong-Hang Zhang

**Affiliations:** 1 College of Engineering, Huaqiao University, Quanzhou 362021, China; 2 College of Information Science and Engineering, Huaqiao University, Xiamen 361021, China

**Keywords:** density functional theory, geometrical structure, stability, density of states, magnetic properties

## Abstract

The geometrical structures, relative stabilities, and electronic and magnetic properties of niobium carbon clusters, Nb_7_C_n_ (n = 1–7), are investigated in this study. Density functional theory (DFT) calculations, coupled with the Saunders Kick global search, are conducted to explore the structural properties of Nb_7_C_n_ (n = 1–7). The results regarding the average binding energy, second-order difference energy, dissociation energy, HOMO-LUMO gap, and chemical hardness highlight the robust stability of Nb_7_C_3_. Analysis of the density of states suggests that the molecular orbitals of Nb_7_C_n_ primarily consist of orbitals from the transition metal Nb, with minimal involvement of C atoms. Spin density and natural population analysis reveal that the total magnetic moment of Nb_7_C_n_ predominantly resides on the Nb atoms. The contribution of Nb atoms to the total magnetic moment stems mainly from the 4d orbital, followed by the 5p, 5s, and 6s orbitals.

## 1. Introduction

The research on nanoclusters represents a significant branch within the fields of nanoscience and nanotechnology, providing a potent pathway for crafting tailored nanomaterials characterized by unique physical and chemical attributes. Consequently, significant advancements have been achieved in this field over the past few decades [1,2,3,4,5,6,7]. Transition metal-carbide clusters possess unique electronic and magnetic properties that make them promising candidates for various applications, ranging from nanoelectronics to energy storage. By delving into the structures, stabilities, and electronic and magnetic properties of transition metal–carbon clusters, researchers can unravel the underlying principles governing the behavior of transition metal-carbide systems at the nanoscale. Moreover, the investigation of transition metal–carbon clusters offers insights into the intricate interplay between metal and carbon atoms within the cluster framework. The coordination environment of metal atoms with carbon atoms influences not only the structural stability but also the electronic and magnetic properties of the clusters. Understanding how these interactions manifest at the atomic level is crucial for designing nanomaterials with tailored functionalities. Niobium carbon clusters, Nb_n_C_m_ clusters, have been the subject of significant interest in recent years due to their potential applications in numerous areas including catalysis, optoelectronics, and materials science [8,9,10,11,12,13,14]. With niobium’s versatile oxidation states and carbide’s decisive role in determining cluster stability and reactivity, understanding the behavior of niobium carbide clusters is essential and could enable the design of advanced materials and catalysts with specific properties. Furthermore, the study of niobium carbon clusters can also contribute to the broader understanding of transition metal-carbide clusters.

Various experimental techniques, such as photoelectron spectroscopy [15,16,17,18], mass spectroscopy [19,20,21,22], X-ray diffraction spectroscopy [17,23], and infrared absorption spectroscopy [24], have been employed to explore the physical and chemical properties of niobium carbides and their ions. Wang and co-workers investigated a series of mono-niobium carbide clusters, NbC_n_^−^ (n = 2–7), using anion photoelectron spectroscopy [16]. The linear NbC_n_^−^ were observed to have high electron binding energies and exhibited an even–odd alternation, similar to that observed for pure linear carbon clusters in the same size range. NbC_2_^−^ and NbC_3_^−^ were shown to have C_2v_ cyclic structures. The mass spectroscopic studies conducted by Duncan and co-workers [19] as well as Castleman and co-workers [20] have revealed that the smaller Nb_m_C_n_^+^ clusters, especially for n = 2–4, are particularly stable. Jarrold and co-workers reported experimental studies of the structures of gas-phase NbC_n_^+^ (n = 28–50) clusters. The experiments, which use injected-ion drift-tube techniques, indicate that for fullerenes containing an even number of carbon atoms, the niobium metal is endohedral, but for fullerenes with an odd number of carbon atoms, the niobium metal is bound as part of the carbon cage [25]. Mafuné and co-workers determined ionization energies for a series of Nb_n_C_m_ (n = 3–10, m = 0–7) clusters [21].

Theoretical studies primarily focus on employing density functional theory (DFT) calculations to explore the geometric and electronic structures of pure niobium clusters and niobium carbon clusters. This provides crucial insights into their stability, reactivity, and electronic properties, which plays a pivotal role in customizing these clusters to meet specific application requirements. A theoretical investigation of structures, spectra, and energies of niobium clusters from Nb_13_ to Nb_20_ is performed by Pham and Minh [26]. The Nb_15_ system is observed to be stable and it can form a highly symmetric structure in all charged states with both open and closed electron shells. The geometric and electronic structures of niobium carbon clusters, ranging in size from [Nb_2_C_2_] to [Nb_12_C_20_], were discussed by Dance and Harris [27]. Vertical ionization energies and normalized binding energies are provided for all isomers. The species [Nb_8_C_12_] and [Nb_14_C_13_] have the same structures as the Ti analogs. Balasubramanian and co-workers determined the geometric and electronic structures of NbC_n_ (n = 3–8), and equilibrium geometries, Gibbs free energies, and heat capacity functions as a function of temperatures were computed [28].

In this paper, we present the theoretical investigation of a series of niobium carbon clusters, Nb_7_C_n_ (n = 1–7). The geometric structures are predicted by employing the Saunders ‘Kick’ (SK) global search method combined with DFT calculation. To better understand how the Nb and C atoms affect the molecular orbitals, we plot the total density of states (TDOS) and the partial density of states (PDOS) of Nb_7_ and C_n_. Meanwhile, the magnetic properties are discussed in detail.

## 2. Results and Discussion

### 2.1. Structure of Nb_7_C_n_ (n = 1–7)

**Figure 1:**
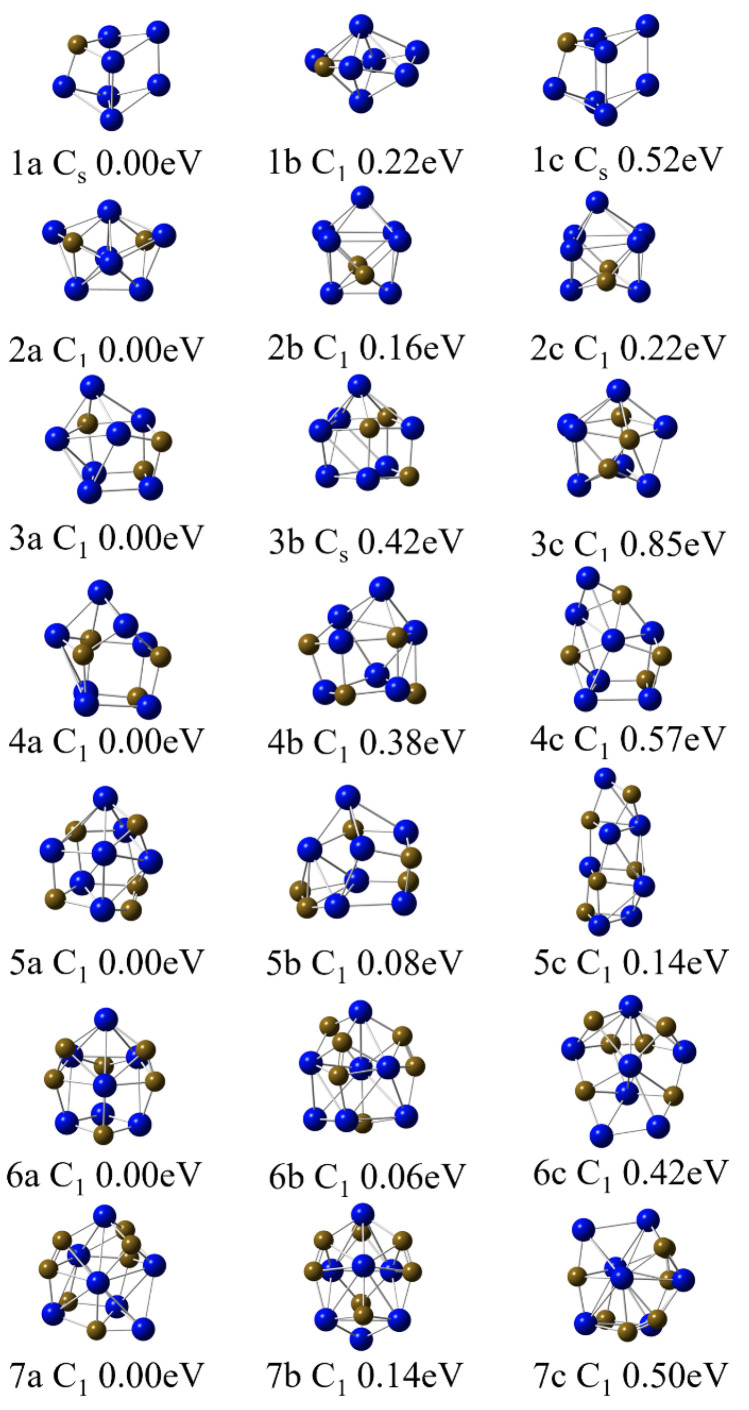
Low-energy isomers of Nb_7_C_n_ (n = 1–7) clusters.

The lowest-energy structure and low-lying isomers of Nb_7_C_n_ (n = 1–7) at the B3LYP/Nb/SDD//C/6−311+G(2d) level are listed in Figure 1. The Cartesian coordinates of the lowest-energy structures of Nb_7_C_n_ (n = 1–7) are summarized in Table S1 of the Supplementary Materials. The two criteria for selecting the ground-state structure of clusters in this study are (1) the principle of lowest energy and (2) the agreement between theoretical predictions and experimental results of vertical ionization potential (VIP). The ground-state structures of the Nb_7_C_n_ series exhibit an outer body structure. It is predicted that as the number of C atoms increases, the structures of Nb_7_C_n_ clusters gradually tend towards hollow spherical structures. In addition, C atom aggregation areas will appear on the surface of the structure in the subsequent structural evolution process.

The lowest-energy structure 1a of the Nb_7_C cluster is a distorted tetragonal prism in which carbon atoms occupy one corner of the quadrangle. The point group symmetry is C_s_ and the spin state is a doublet state. Structure 1b exhibits a twisted hexagonal bipyramid-shaped structure higher in energy than the ground-state structure by 0.22 eV. The quartet-state structure 1c is higher in energy than doublet state 1a by 0.52 eV. The low-energy isomers of the Nb_7_C_2_ cluster all exhibit C_1_ symmetry. Among them, 2a adopts a twisted bipyramidal shape, while 2b and 2c are three-dimensional structures formed by splicing three twisted pyramids, with relative energies of 0.16 eV and 0.22 eV, respectively. Moving on to the Nb_7_C_3_ cluster, the three C atoms in 3a attach to the outer side of the C_1_ symmetric isomer, resulting in an irregular three-dimensional shape. Isomers 3b and 3c have relative energies 0.42 eV and 0.85 eV higher than that of 3a, respectively. The lowest-energy isomer 4a of Nb_7_C_4_ can be viewed as that obtained from the capping of a C atom to the lowest-energy structure of Nb_7_C_3_. Isomers 4b and 4c are less stable than 4a by 0.38 and 0.57 eV, respectively. The Nb_7_C_5_ system exhibits three isomers with 5a being the ground-state structure. Isomer 5b is only 0.08 eV higher than that of isomer 5a. Within the expected accuracy of the methods, isomers 5a and 5b can be regarded as nearly degenerate. In this case, we calculate the VIPs of both isomers 5a and 5b. The calculated VIPs of 5a and 5b are 4.86 and 5.14 eV, respectively. Compared with the results of isomers 5a and 5b, the calculated VIP value for structure 5a obviously agrees better with the experimental result of 4.7 ± 0.1 eV. These results indicate that isomer 5a is the most stable isomer in current DFT calculations of the Nb_7_C_5_ cluster. Isomer 5c is less stable than 5a by 0.14 eV. As for the neutral Nb_7_C_6_ cluster, the energy gap of isomers 6a and 6b is 0.06 eV. Given the small energy difference between structures 6a and 6b, we also performed VIPs calculations for both the isomers. The results found that the calculated VIP for isomer 6a (5.08 eV) is in better agreement with the experimental values (4.91 eV) than that for isomer 6b (5.16 eV). Therefore, isomer 6a should be the ground-state structure. In the doped Nb_7_C_7_ cluster, an equal number of Nb and C atoms does not result in a uniform distribution but instead leads to the aggregation of carbon atoms. This phenomenon results in shorter C-C bond lengths and greater bond energies, further enhancing the stability of the structure. Compared to the 7b isomer with a more uniform atomic distribution, the energy is 0.14 eV lower. The energy of 7c is 0.50eV higher than that of the ground-state structure.

Based on the lowest-energy structures, VIPs are calculated and compared with the experimental values, and the results are shown in Table 1. The vertical ionization potential is defined as VIP = E (cation at optimized neutral geometry) − E (optimized neutral geometry). From Table 1, we can see that the calculated VIPs are all in good agreement with the experimental results. The calculated VIP values of the lowest-energy structures 1a (5.16 eV), 2a (4.72 eV), 3a (4.78 eV), 4a (4.76 eV), 5a (4.86 eV), 6a (5.08 eV), and 7a (5.19 eV) agree well with the experimental values of 5.20 ± 0.08, 4.7 ± 0.1, 4.7 ± 0.08, 4.75 ± 0.07, 4.7 ± 0.1, 4.91 ± 0.07, and 5.1 ± 0.1 eV [21], respectively.

The infrared (IR) spectra of the lowest-energy structures of Nb_7_C_n_ (n = 1–7) clusters are computed based on DFT calculations. The calculated spectra are plotted in Figure S1 of the Supporting Information (ESI). Based on Figure S1, the IR spectra of Nb_7_C_n_ (n = 1–7) clusters cover the range of 0 to 2000 cm^−1^. We applied a frequency scaling factor of 0.9692 [29], determined at the B3LYP/6−311G(2d) level, to adjust the IR spectra during plotting. From Figure S1, it is evident that the IR spectra of Nb_7_C_n_ (n = 1–4) clusters span from 0 to 800 cm^−1^. However, for Nb_7_C_n_ (n = 5–7) clusters, a noticeable peak emerges within the range of 1200–1400 cm^−1^. In the case of Nb_7_C clusters, two distinct and strong spectral peaks are found around 600 and 700 cm^−1^, alongside some weaker peaks below 250 cm^−1^. For Nb_7_C_2_, the highest peak is centered at around 600 cm^−1^, and there are some minor peaks within the range of 100–400 cm^−1^. The highest-intensity peak of Nb_7_C_3_ is observed at around 670 cm^−1^, with several lower-intensity peaks falling between 100 and 350 cm^−1^. For Nb_7_C_4_, we mainly distinguish one broad band ranging from 450 to 750 cm^−1^, and the broadness of the peak suggests that it originates from multiple vibrational modes. The spectra of Nb_7_C_5_ and Nb_7_C_6_ clusters exhibit an intense peak at around 1300 cm^−1^, with some lower-intensity peaks below 800 cm^−1^. The spectrum for Nb_7_C_7_ contains two distinguishable peaks around 1250 and 1350 cm^−1^, as well as a wide band below 800 cm^−1^ with a maximum around 600 cm^−1^.

### 2.2. Stability

**Figure 2:**
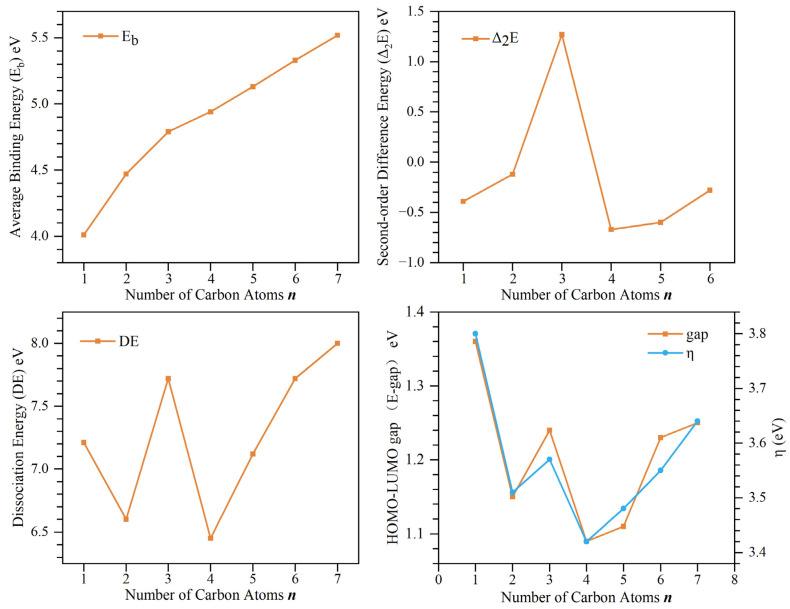
The average binding energy (E_b_), second-order difference energy (Δ_2_E), dissociation energy (DE), HOMO-LUMO gap (E-gap), and chemical hardness (η) for Nb_7_C_n_ (n = 1–7) clusters.

To explore the stability of the ground-state structure of Nb_7_C_n_ (n = 1–7) clusters, the average binding energy (E_b_), second-order difference energy (Δ_2_E), dissociation energy (DE), HOMO-LUMO gap (E-gap), and chemical hardness (η) of the lowest-energy isomers are calculated in Table 1 and plotted in Figure 2. They are defined as follows:

E_b_(Nb_7_C_n_) = [7E(Nb) + nE(C) − E(Nb_7_C_n_)]/(7 + n) (1)

Δ_2_E(Nb_7_C_n_) = E(Nb_7_C_n+1_) + E (Nb_7_C_n−1_) − 2E(Nb_7_C_n_) (2)

DE(Nb_7_C_n_) =E(Nb_7_C_n−1_) + E(C) − E (Nb_7_C_n_) (3)

E-gap = ε(LUMO) − ε(HOMO) (4)

η = VIP − VEA (5)

where E(Nb) represents the energy of a single Nb atom, E(C) represents the energy of a single C atom, and ε(HOMO) and ε(LUMO) represent the energy of the highest occupied orbit and the energy of the lowest empty orbit of the ground-state structure of Nb_7_C_n_ (n = 1–7) clusters, respectively.

As shown in Figure 2, the average binding energies of the ground-state structure of Nb_7_C_n_ (n = 1–7) clusters generally show an increasing trend, with an obvious slope change at n = 3, which indicates that isomer Nb_7_C_3_ has excellent thermal stability among Nb_7_C_n_ (n = 1–7) clusters. This corresponds to the high second-order differential energy and dissociation energy of the isomer Nb_7_C_3_. Chemical hardness and HOMO-LUMO gap represent the degree of response of the molecule to the outside world, especially the addition or removal of electrons under the action of external potential fields. The overall change trend of E-gap and η, which reflect the chemical stability of the ground-state structure of the Nb_7_C_n_ cluster, is the same. The E-gap and η values of isomer Nb_7_C_1_-a are highest among all ground-state isomers, indicating that its chemical stability is higher than those of other isomers. In addition, when the number of carbon atoms n > 4, as the number of incorporated carbon atoms increases, the energy indicators of the cluster increase monotonically. This is because Nb atoms can form Nb-C bonds by sharing electrons with C atoms. Moreover, owing to the lower energy levels of Nb’s d orbitals, they can donate electrons to the p orbitals of the C atoms, thereby reinforcing the Nb-C bonds. Additionally, the distance between Nb and C atoms is shorter compared to that between Nb atoms, indicating a stronger van der Waals interaction between Nb and C atoms, thereby enhancing the stability of the present clusters.

Among all ground-state isomers of Nb_7_C_n_ (n = 1–7) clusters, isomers Nb_7_C_3_ and Nb_7_C_4_ show obvious differences in stability. Isomer Nb_7_C_3_ shows a local maximum value among the ground-state isomers of all sizes of Nb_7_C_n_ (n = 1–7) clusters in the curves of Δ_2_E, DE, E-gap, and η shown in Figure 2. This observation suggests that Nb_7_C_3_ possesses higher thermodynamic and chemical stability, making it a potential candidate for a magic cluster. In contrast, minima appeared at n = 4, suggesting that isomer 4a has poor thermal and chemical stability and can be used as a potential detector material and chemical reaction indicator.

### 2.3. Density of States

**Figure 3:**
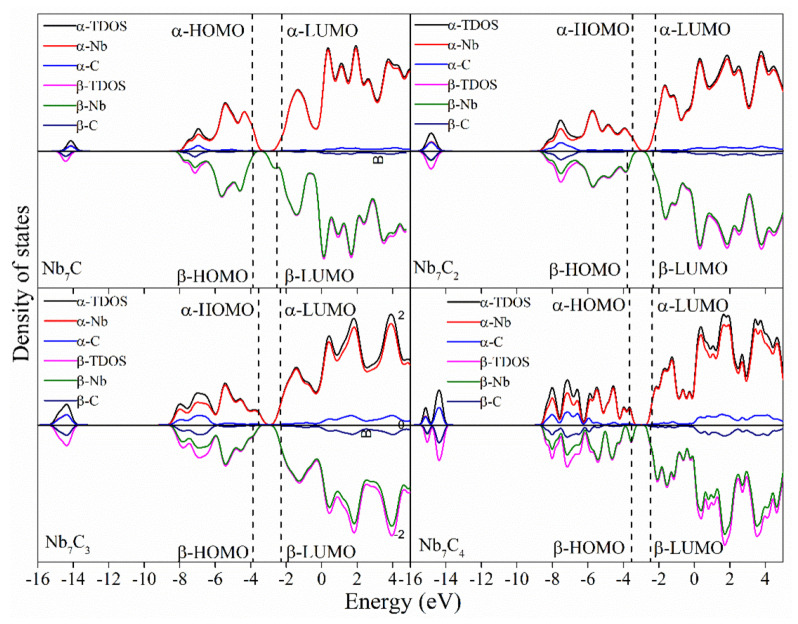
The total density of states (TDOS) and partial density of states (PDOS) of the Nb_7_C_n_ (n = 1−4) with a full−width at half−maximum (FWHM) of 0.5 eV.

**Figure 4:**
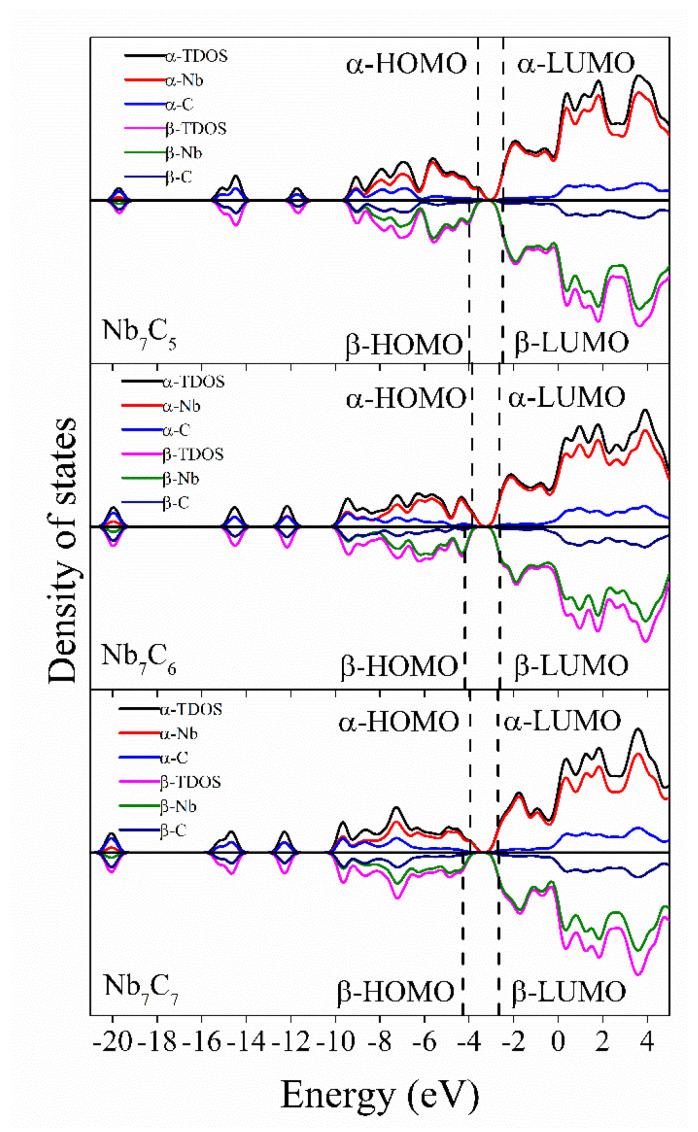
The total density of states (TDOS) and partial density of states (PDOS) of the Nb_7_C_n_ (n = 5−7) with a full−width at half−maximum (FWHM) of 0.5 eV.

To further explore how Nb and C atoms affect the molecular orbital and HOMO-LUMO energy gap of the Nb_7_C_n_ (n = 1–7) clusters, the density of states diagram of the ground-state structures of the Nb_7_C_n_ (n = 1–7) clusters is shown in Figure 3 and Figure 4. Analysis of the total density of states (TDOS) plot for the lowest-energy structures of the Nb_7_C_n_ (n = 1–7) clusters reveal a negligible disparity in profile between the alpha-TDOS and beta-TDOS, thereby signifying a minimal degree of spin polarization. To offer a more nuanced evaluation of the contribution of Nb and C atoms to the total density of states, respectively, we plotted the partial density of states (PDOS) of Nb_7_ and C_n_, respectively. Compared to the PDOS curves of Nb, the PDOS profiles of the C atoms generally exhibit lower magnitudes. At the HOMO and LUMO positions, the PDOS of the Nd fragment is very close to the TDOS. As the number of C atoms increases, a distinct aggregation phenomenon of carbon atoms is observed within the structure of the doped cluster. Upon comparing the density of states plots of Nb_7_C_n_ clusters (Figure 3 and Figure 4), it becomes evident that the contribution of carbon atoms to the frontier orbitals demonstrates a trend of localized enhancement to some degree. We provide the compositions of the frontier molecular orbitals (alpha-HOMO, alpha-LUMO, beta-HOMO, beta-LUMO) for Nb_7_C_n_ (n = 1–7) in Table 2.

It can be seen in Table 2 that the percentage contribution of Nb in the front molecular orbitals is very high and far exceeds that of the C atom. For α-HOMO, Nb atoms contribute approximately from 88.95% to 99.45% to the Nb_7_C_n_ clusters. For α-LUMO, Nb atoms contribute approximately from 91.86% to 98.8%. For β-HOMO, Nb atoms contribute approximately from 84.35% to 98.88%. For β-LUMO, Nb atoms contribute approximately from 92.29% to 99.87%. This indicates that HOMO and LUMO are mainly composed of orbitals of the transition metal Nb, with a weak involvement of C atoms. Such an observation suggests the pivotal role played by Nb atoms in modulating the electronic structure within these isomers of Nb_7_C_n_ (n = 1–7) clusters.

### 2.4. Magnetic Properties

**Figure 5:**
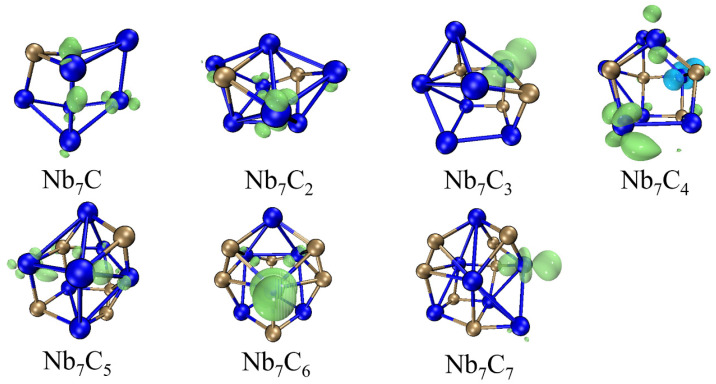
The spin density isosurfaces of lowest-energy structures of Nb_7_C_n_ (n = 1–7). The isosurface is set to ±0.01. The green and blue isosurfaces show that the spin density has positive and negative values, respectively.

The magnetic moment is an important concept in physics, chemistry, and materials science, as it plays a significant role in understanding and controlling magnetic properties and phenomena. The spin density ($\rho_{alpha}-\rho_{beta}$) refers to the distribution of unpaired electrons with different spin orientations within a molecule or material. It provides information about the electronic structure and magnetic properties of the system. We utilized Multiwfn in conjunction with the VMD program to visualize the spin-density isosurfaces of the lowest-energy structures of Nb_7_C_n_ (n = 1–7), as shown in Figure 5. The total magnetic moments of Nb_7_C_n_ (n = 1–7) as well as the local magnetic moments of Nb atoms and C atoms in Nb_7_C_n_ clusters are presented in Table 3.

In Figure 5, the spin density diagram shows that the excess unpaired electrons are predominantly distributed around the Nb atoms. In Table 3, we can find that Nb_7_C_n_ clusters have total magnetic moments of 1 μ_B_ and the total magnetic moment is mainly located on the Nb atoms, whereas the magnetic moment located on the C atoms is almost negligible. To further understand the magnetic properties of Nb_7_C_n_ clusters, we performed a detailed analysis of the local magnetic moment of Nb atoms in Nb_7_C_n_ clusters by natural population analysis calculations. Table 3 shows that for the Nb_7_C and Nb_7_C_2_ clusters, the magnetic moment of Nb atoms is mainly from the 4d orbital, followed by the 5s and 5p orbitals with a small contribution to the magnetic moment of the Nb atom. For the other Nb_7_C_n_ (n = 1–7), the magnetic moment of Nb atoms still comes mainly from the 4d orbital, followed by 5p and 6s orbitals. For the Nb_7_C_5_ cluster, the contribution of 5p, 6s, and 6p orbitals to the magnetic moment of Nb atom is nearly equal. The contribution of C atoms to the total moment is minimal and mainly originates from the 2p orbitals.

## 3. Computational Methods

Global minimum searches for low-lying structures of Nb_7_C_n_ (n = 1–7) clusters were performed in three steps: Firstly, we employed the SK global search method [30,31,32] combined with DFT calculation to search and optimize the structure. In recent years, our research group has effectively predicted the ground-state structures and electronic properties of binary mixed clusters utilizing the SK-DFT method [33,34,35,36,37,38,39,40,41]. All the atoms are placed at the same point initially and then are “kicked” randomly with a sphere of some radius. The Kick method runs approximately 500 times at the B3LYP functional [42,43] using the 3–21 G [44] basis set until no new minima appear. Secondly, the isomers were ranked according to their total energy at the B3LYP/3-21G level. Thirdly, several pertinent lower-lying isomers were chosen for additional optimization employing the triply split basis set with polarization and diffuse functions. Specifically, the 6–311+G(2d) [45] basis set was employed for C. For the heavier atoms, previous studies [46,47,48,49] have confirmed the significance of relativistic effects in simulating the properties of systems involving heavy atoms. Therefore, using appropriate relativistic treatment methods is important when dealing with systems containing heavy atoms. The SDD pseudopotential basis set [50], which accounts for relativistic effects, was used for Nb. The interaction between valence electrons and the inert core is included in the pseudopotential. This allows us to reduce computational times and provide reliable calculations of geometry and electronic properties. Geometries are regarded as completing the optimization when the maximum force, the root-mean-square (RMS) force, the maximum displacement of atoms, and the RMS displacement of atoms have magnitudes less than 0.00045, 0.0003, 0.0018, and 0.0012 a.u., respectively. The self-consistent field convergence criterion was set as 10^−8^. Structural optimization with frequency analysis were considered at the same time. The image frequency would be eliminated until every structure optimized had no image frequency to confirm that the low-lying isomers were local minima. All calculations were performed with the GAUSSIAN09 program [51]. Density of states (DOS) and orbital composition analysis were performed by Multiwfn program [52] and visualized by Visual Molecular Dynamics (VMD 1.9.3) software [53].

## 4. Conclusions

We have investigated the structural, electronic, and magnetic properties of Nb_7_C_n_ (n = 1–7) clusters by employing density functional theory calculations. The relative stability analysis indicates that Nb_7_C_3_ is more stable than the other clusters, as it exhibits a higher second-order difference energy, dissociation energy, HOMO-LUMO gap, and chemical hardness. In contrast, Nb_7_C_4_ is less stable, as it shows lower values of these stability indicators. The composition of the frontier molecular orbitals reveals that the predominant contribution originates from Nb atoms, accounting for 84.35% to 99.87% of the molecular orbital. The total magnetic moment of Nb_7_C_n_ (n = 1–7) is mainly located on the Nb atoms, whereas the magnetic moment located on the C atoms is almost negligible. For the Nb atoms, the 4d orbit contributes most to the total magnetic moment, followed by the 5p, 5s, and 6s orbits.

## Figures and Tables

**Table 1:** The vertical ionization potential (VIP), average binding energy (E_b_), second-order difference energy (Δ_2_E), dissociation energy (DE), HOMO-LUMO gap (E-gap), and chemical hardness (η) for Nb_7_C_n_ (n = 1–7) clusters; all energies are in eV.

Isomers	VIP		E_b_	Δ_2_E	DE	E-Gap	η
	Calc.	Expt. [21]					
Nb_7_C	5.16	5.20 ± 0.08	4.01	−0.39	7.21	1.36	3.80
Nb_7_C_2_	4.72	4.7 ± 0.1	4.47	−0.12	6.60	1.15	3.51
Nb_7_C_3_	4.78	4.7 ± 0.08	4.79	1.27	7.72	1.24	3.57
Nb_7_C_4_	4.76	4.75 ± 0.07	4.94	−0.67	6.45	1.09	3.42
Nb_7_C_5_	4.86	4.7 ± 0.1	5.13	−0.60	7.12	1.11	3.48
Nb_7_C_6_	5.08	4.91 ± 0.07	5.33	−0.28	7.72	1.23	3.55
Nb_7_C_7_	5.19	5.1 ± 0.1	5.52		8.00	1.25	3.64

**Table 2:** The compositions of the frontier molecular orbitals for Nb_7_C_n_ (n = 1–7).

Isomers	Atoms	α-HOMO	α-LUMO	β-HOMO	β-LUMO
Nb_7_C	Nb	98.72%	98.80%	98.68%	99.87%
	C	1.28%	1.20%	1.32%	0.13%
Nb_7_C_2_	Nb	99.45%	97.73%	98.88%	99.47%
	C	0.55%	2.27%	1.12%	0.53%
Nb_7_C_3_	Nb	94.32%	96.29%	92.69%	96.63%
	C	5.68%	3.71%	7.31%	3.37%
Nb_7_C_4_	Nb	91.37%	95.49%	96.44%	92.42%
	C	8.63%	4.51%	3.56%	7.58%
Nb_7_C_5_	Nb	88.95%	92.15%	88.60%	92.54%
	C	11.05%	7.85%	11.40%	7.46%
Nb_7_C_6_	Nb	93.80%	94.57%	84.35%	94.29%
	C	6.20%	5.43%	15.65%	5.71%
Nb_7_C_7_	Nb	94.49%	91.86%	84.66%	92.29%
	C	5.51%	8.14%	15.34%	7.71%

**Table 3:** Total magnetic moments of Nb_7_C_n_ clusters and local magnetic moments for the C atom and magnetic moments of 4d, 5s, 5p, 6s, and 6p orbitals for the Nb atoms; all units in μ_B_.

Isomers	Nb					C	Total
	5s	4d	5p	6s	6p		
Nb_7_C	0.15	0.74	0.12	0.00	−0.01	−0.04	1
Nb_7_C_2_	0.12	0.63	0.20	0.00	0.00	−0.02	1
Nb_7_C_3_	0.01	0.75	0.14	0.12	0.00	−0.01	1
Nb_7_C_4_	0.03	0.47	0.24	0.08	0.00	0.12	1
Nb_7_C_5_	0.02	0.36	0.18	0.14	0.15	0.16	1
Nb_7_C_6_	0.05	0.74	0.08	0.07	0.02	0.02	1
Nb_7_C_7_	0.00	0.66	0.13	0.14	0.05	0.02	1

## Data Availability

The data presented in this study are available on request from the corresponding authors.

## Supplementary Materials

The following supporting information can be downloaded at: https://www.mdpi.com/article/10.3390/molecules29081692/s1, Table S1: Cartesian coordinates for the lowest energy structure of Nb_7_C_n_ (n = 1−7) at the B3LYP/Nb/ SDD//C/6−311+G(2d) level. Figure S1: The IR spectra of the lowest energy structures of Nb_7_C_n_ (n = 1−7) clusters.
