# Supplementary material for: Investigation of Structures, Stabilities, and Electronic and Magnetic Properties of Niobium Carbon Clusters Nb7Cn (n = 1–7)
Source: Molecules. 2024 Apr 9;29(8):1692. doi: 10.3390/molecules29081692 (PMC11051814; doi:10.3390/molecules29081692)
Supplement: Supplementary file 1 [file molecules-29-01692-s001.zip › molecules-2911827-supplementary.pdf]

Supplementary Materials

Investigation of Structures, Stabilities, and Electronic and Magnetic Properties of Niobium Carbon Clusters Nb7Cn (n = 1–7)

Hui-Fang Li [1], Huai-Qian Wang [1,2,*], Jia-Ming Zhang [2], Lan-Xin Qin [1], Hao Zheng [2] and Yong-Hang Zhang [2]

1 College of Engineering, Huaqiao University, Quanzhou 362021, China
2 College of Information Science and Engineering, Huaqiao University, Xiamen 361021, China
* Correspondence: hqwang@hqu.edu.cn

Table of Contents

Table S1. Cartesian coordinates for the lowest energy structure of Nb_7C_n (n =1-7) at the B3LYP/Nb/SDD//C/6-311+G(2d) level.

Figure S1. The IR spectra of the lowest-energy structures of Nb_7C_n (n=1-7) clusters.

Table S1. Cartesian coordinates for the lowest energy structure of Nb_7C_n (n =1-7) at the B3LYP/Nb/SDD//C/6-311+G(2d) level.

Nb_7C

Atom	X	Y	Z
Nb	1.768834	-0.519511	0.000000
Nb	-0.254648	-1.566425	1.347622
Nb	-2.199216	-0.592950	0.000000
Nb	-0.254648	-1.566425	-1.347622
Nb	-0.254648	1.094167	1.467610
Nb	1.692680	1.866832	0.000000
Nb	-0.254648	1.094167	-1.467610
C	-1.665345	1.299325	0.000000

Nb_7C_2

Atom	X	Y	Z
Nb	1.077880	1.419154	0.735389
Nb	-1.077459	1.430304	-0.715138
Nb	-2.341355	-0.785714	-0.535413
Nb	1.302786	0.289362	-1.661125
Nb	-0.000181	-1.681397	-0.013960
Nb	-1.302429	0.263758	1.665734
Nb	2.340796	-0.794484	0.525657
C	-0.583169	-0.468397	-1.681930
C	0.582914	-0.494987	1.674106

Nb_7C_3

Atom	X	Y	Z
Nb	-0.536203	-0.232820	-1.801387
Nb	1.183527	1.549572	-0.715415
Nb	-1.136462	1.674189	0.269735
Nb	-2.425165	-0.414021	0.048646
Nb	-0.298637	-1.887401	0.675911
Nb	0.924015	0.550235	1.822212
Nb	2.119147	-0.799107	-0.548089
C	0.549417	-1.813515	-1.233086
C	1.615534	-1.223451	1.342875
C	-1.004803	0.025874	1.587516

Nb_7C_4

Atom	X	Y	Z
Nb	1.057318	-0.26099	1.736534
Nb	-1.55844	1.583457	0.597334
Nb	1.26285	1.714998	-0.41153
Nb	2.533946	-0.42147	-0.2882
Nb	0.187106	-1.94918	-0.65554
Nb	-1.13171	0.48085	-1.68288
Nb	-2.2018	-0.92498	0.706031
C	-0.40813	-1.6255	1.264964
C	-1.75707	-1.38392	-1.16805
C	0.810577	-0.11018	-1.46423
C	0.334662	1.597935	1.355294

Nb_7C_5

Atom	X	Y	Z
Nb	-0.70951	-0.05709	-1.68426
Nb	2.016795	0.958266	-0.79061
Nb	-0.55522	2.16208	0.353432
Nb	-2.4324	0.297022	0.127084
Nb	-0.75958	-1.58325	1.087583
Nb	1.383634	0.336634	1.621099
Nb	1.322892	-1.6271	-0.6642
C	-0.75394	-2.23107	-1.05442
C	1.292109	-1.65517	1.343759
C	-0.71458	0.477281	1.517641
C	0.30518	1.740497	-1.38962
C	-1.95066	-1.65645	-0.75995

Nb_7C_6

Atom	X	Y	Z
Nb	0.574181	-1.11489	-1.39113
Nb	-2.14377	-1.29241	-0.0392
Nb	0.577441	-1.20807	1.32156
Nb	2.751336	-0.21979	-0.00705
Nb	0.758786	1.767129	0.053577
Nb	-1.48325	0.771245	1.510955
Nb	-1.48195	0.860723	-1.46124
C	0.576189	1.01244	-2.02909
C	-1.3268	2.11169	0.064834

C	1.843188	0.591181	1.65812
C	-0.45505	-2.31203	-0.07194
C	1.844828	0.695168	-1.61967
C	0.573647	0.881293	2.083335

Nb_7C_7

Atom	X	Y	Z
Nb	0.715775	-1.53497	0.962424
Nb	-1.56396	-2.01567	-0.6617
Nb	0.116678	0.136086	-1.42218
C	0.613626	0.342967	2.065242
C	1.885473	0.27982	1.535186
C	0.42756	-2.14272	-1.21044
Nb	-1.37347	0.111295	1.395129
Nb	2.754614	-0.0629	-0.32496
C	1.682281	-1.63881	-1.0944
Nb	-1.82746	1.936903	-0.51685
Nb	0.729494	1.976523	0.487257
C	-2.02544	-0.01674	-0.83975
C	1.830165	1.433963	-1.16059
C	-1.35004	-1.99813	1.257453

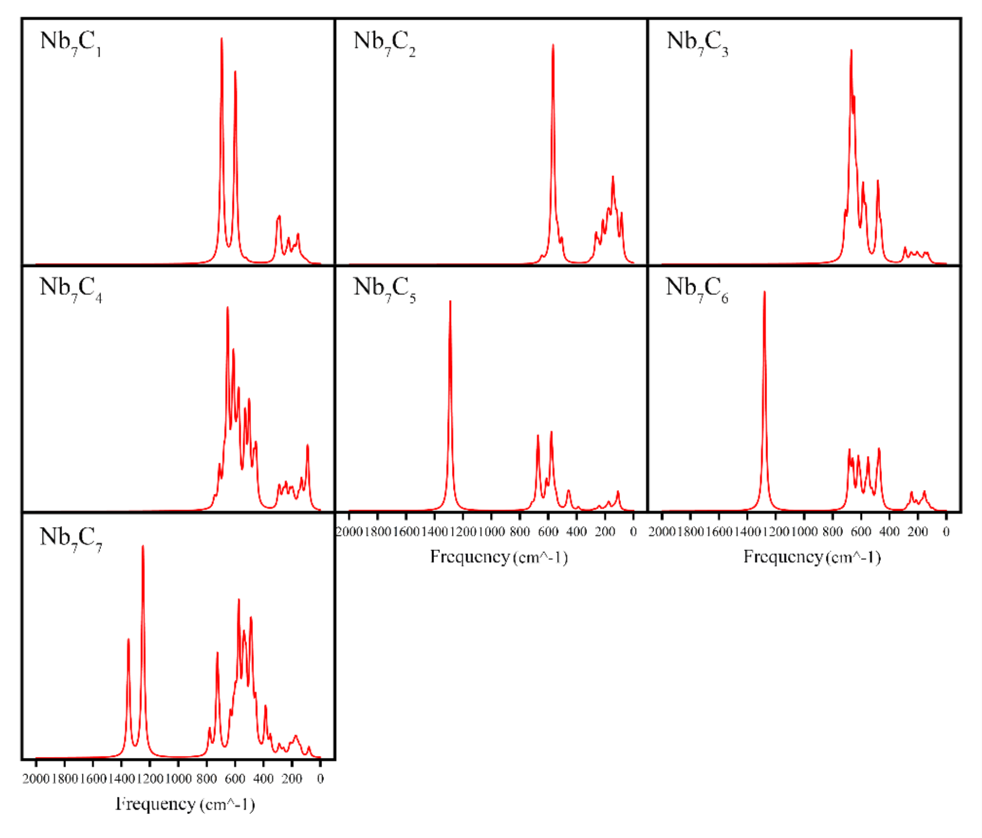

Figure S1. The IR spectra of the lowest-energy structures of Nb_7C_n (n=1-7) clusters.
